# Incidence and mortality of pulmonary embolism in COVID-19: a systematic review and meta-analysis

**DOI:** 10.1186/s13054-020-03175-z

**Published:** 2020-07-27

**Authors:** Shu-Chen Liao, Shih-Chieh Shao, Yih-Ting Chen, Yung-Chang Chen, Ming-Jui Hung

**Affiliations:** 1 Department of Emergency Medicine, Keelung Chang Gung Memorial Hospital, Keelung, Taiwan; 2 College of Medicine, Chang Gung University, Taoyuan, Taiwan; 3 School of Pharmacy, Institute of Clinical Pharmacy and Pharmaceutical Sciences, College of Medicine, National Cheng Kung University, Tainan, Taiwan; 4 Department of Pharmacy, Keelung Chang Gung Memorial Hospital, Keelung, Taiwan; 5 Section of Nephrology, Department of Internal Medicine, Keelung Chang Gung Memorial Hospital, Keelung, Taiwan; 6 School of Medicine, Institute of Public Health, National Yang Ming University, Taipei, Taiwan; 7 Section of Nephrology, Department of Internal Medicine, Linkou Chang Gung Memorial Hospital, Taoyuan, Taiwan; 8 Section of Cardiology, Department of Internal Medicine, Keelung Chang Gung Memorial Hospital, Keelung, Taiwan; 9 Community Medicine Research Center, Keelung Chang Gung Memorial Hospital, Keelung, Taiwan

**Keywords:** Pulmonary embolism, COVID-19, Systematic review, Meta-analysis

Coronavirus disease 2019 (COVID-19) remains an increasing global pandemic, with significant morbidity and mortality. Severe complications of COVID-19 associated with coagulation changes, mainly characterized by increased D-dimer and fibrinogen levels with higher thrombosis risk, in particular pulmonary embolism (PE), have been reported recently [1]. However, the epidemiology of PE among COVID-19 patients is currently only based on small case series and retrospective studies. This systematic review and meta-analysis addresses this gap in knowledge, facilitating first-line healthcare providers’ understanding of PE incidence and mortality in COVID-19.

Relevant Chinese or English language studies were identified by systematic search of EMBASE and PUBMED from inception to June 28, 2020, using the keywords “COVID-19,” “pulmonary embolism,” “incidence,” “prevalence,” and “mortality” with appropriate MeSH terms, whereby the reference lists of identified studies yielded additional sources. We excluded conference abstracts, other types of publications (e.g., editorials, review articles, commentaries and treatment consensus), and studies lacking PE incidence or mortality rate reports. Two reviewers (SCL, SCS) screened the titles and abstracts for relevance, independently assessed the full texts of the screened search results, and drew up a final list of studies for inclusion through discussion and only after reaching full agreement. All statistical analyses were performed using MedCalc (Windows) version 15.0 (MedCalc Software, Ostend, Belgium). Incidence and mortality rates of PE in COVID-19 are represented as proportions with 95% confidence interval (CI), using the random effects model, and displayed as Forest plot. Heterogeneity among the studies was detected by Cochran *Q* test, whereby a *p* value < 0.10 indicated significant heterogeneity. We assessed the proportion of variation in study estimates attributable to heterogeneity through the *I*^2^ statistic.

We excluded 78 out of 97 articles screened: 20 studies were duplicates, 5 were irrelevant, 3 were conference abstracts, 21 were other types of publications, 28 lacked data on PE incidence or mortality, and 1 was published in French. Ultimately, our analysis included 19 articles, mostly from Europe (84%), and we summarize their demographic data in Table 1. Overall, the incidence and mortality rate of COVID-19 patients developing PE was 15.3% (95%: 9.8–21.9) and 45.1% (95%: 22.0–69.4), respectively. Some evidence of statistical heterogeneity among the studies reporting PE incidence (*I*^2^: 92.0%, *p* < 0.001) and mortality (*I*^2^: 78.6%, *p* < 0.001) in COVID-19 was observed (Fig. 1).

**Table 1:** Study characteristics

First author (Year)	Study design	City (country)	Male (%)	Age (median, years)	Settings	PE diagnosis	D-dimer (median, mg/dL)	Prophylactic anticoagulation (%)	Mechanical ventilation (%)	ARDS (%)	Overall mortality (%)
***Asia***											
Wang Y (2020) [2]	RCT (remdesivir group)	Beijing (China)	56	66	Inpatient	NA	NA	NA	7	10	15
Wang Y (2020) [2]	RCT (placebo group)	Beijing (China)	65	64	Inpatient	NA	NA	NA	13	8	13
***America***											
Riker RR (2020) [3]	Case series	Portland (USA)	NA	NA	Inpatient (ICU)	CTPA	NA	NA	100	100	NA
LeBrun DG (2020) [4]	Retrospective cohort	New York (USA)	33	87*	Inpatient (ICU, ward)	NA	NA	NA	33	NA	56
***Europe***											
Wichmann D (2020) [5]	Case series	Hamburg (Germany)	75	73	Mortuary	Autopsy	90.4	33	33	NA	100
Klok FA (2020) [6]	Retrospective cohort	Leiden (Netherlands)	76	64*	Inpatient (ICU)	CTPA	NA	100	NA	NA	22
Llitjos JF (2020) [7]	Retrospective cohort	Pairs (France)	77	68	Inpatient (ICU)	CDU	1.8	31	100	81	12
Helms J (2020) [8]	Prospective cohort	Strasbourg (France)	81	63	Inpatient (ICU)	CTPA	2.3	100	100	100	9
Menter T (2020) [9]	Retrospective cohort	Basel (Switzerland)	81	76*	Mortuary	Autopsy	4.0	NA	30	NA	100
Florian Bompard (2020) [10]	Retrospective cohort	Paris (France)	70	64	Inpatient, outpatient	CTPA	1.6	53	13	NA	12
Hékimian G (2020) [11]	Retrospective cohort	Paris (France)	NA	NA	Inpatient (ICU)	CTPA or autopsy	NA	NA	NA	NA	NA
Artifoni M (2020) [12]	Retrospective cohort	Nantes (France)	61	64	Inpatient (ICU, ward)	CTPA	0.8	99	11	NA	NA
Fraissé M (2020) [13]	Retrospective cohort	Argenteuil (France)	79	61	Inpatient (ICU)	CDU	2.4	47	89	NA	41
Thomas W (2020) [14]	Retrospective cohort	Cambridge (UK)	69	20–29: 2% 30–39: 5% 40–49: 13% 50–59: 29% 60–69: 22% 70–79: 27% 80–89: 3%	Inpatient (ICU)	CTPA	0.4	NA	83	NA	16
Lodigiani C (2020) [15]	Retrospective cohort	Milano (Italy)	68	66	Inpatient (ICU, ward)	CTPA	Survivors: Day 1–3: 0.4 Day 4–6: 0.4 Day 7–9: 0.5 Non-survivors: Day 1–3: 0.9 Day 4–6: 0.9 Day 7–9: 1.5	79	NA	NA	26
Poissy J (2020) [16]	Case series	Lille (France)	NA	NA	Inpatient (ICU)	CTPA	NA	NA	63	63	14
Gervaise A (2020) [17]	Retrospective cohort	Saint Mande Cedex (France)	75	62*	Outpatient	CTPA	3.6*	NA	57	NA	15
Longchamp A (2020) [18]	Case series	Sion (Switzerland)	64	68*	Inpatient	CTPA	2.1	96	92	NA	20
Leonard-Lorant I (2020) [19]	Retrospective cohort	Strasbourg (France)	66	64	Inpatient (ICU, ward)	CTPA	PE: 15.4 Non-PE: 1.9	46	NA	NA	NA
Grillet F (2020) [20]	Retrospective cohort	Besancon (France)	70	66*	Inpatient (ICU, ward)	CTPA	NA	NA	34	NA	NA

*In studies not reporting the median, results are represented by the mean

*CDU* complete duplex ultrasound, *CTPA* CT pulmonary angiography, *ICU* intensive care unit, *NA* not available, *PE* pulmonary embolism, *RCT* randomized controlled trial

**Fig. 1:**
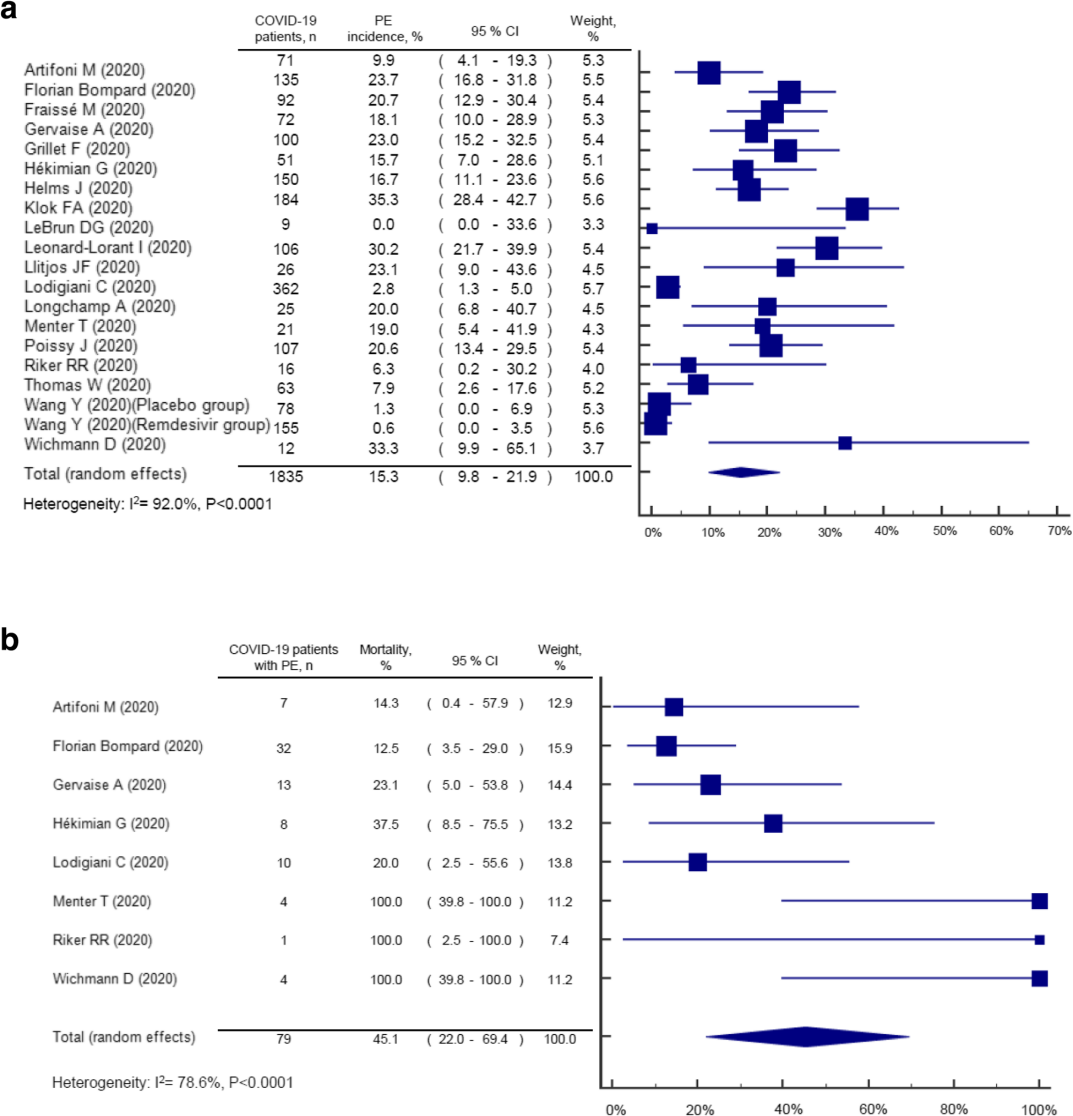
Forest plot of PE incidence and mortality in COVID-19 infections from included studies. **a** PE incidence in COVID-19 infections. **b** PE mortality in COVID-19 infections

With increasing reports of PE following COVID-19 infection, our findings indicate that nearly 2 in 10 developed PE among a total of 1835 COVID-19 patients. Immobilization, inflammation, activated coagulation, and suppressed fibrinolysis have been proposed to explain the occurrence of PE in COVID-19 patients; however, the incidence of PE in COVID-19 patients is higher than in patients with seasonal and pandemic influenza (3%) [21]. In addition, our report indicates COVID-19 patients with PE may have up to 45% higher mortality rate compared to general cases (in-hospital mortality rate 4%) [22]. Therefore, first-line healthcare providers should be vigilant about the occurrence of severe and potentially fatal PE complications in COVID-19 patients [23].

As far as we know, this systematic review is the first summarizing PE incidence and mortality in COVID-19 patients. However, caution is advised in interpreting our findings. First, most published literatures are observational studies, making it difficult to confirm causality between COVID-19 and PE. Second, clinical heterogeneity between studies is noteworthy; for example, the included studies apply different diagnostic tools of varying sensitivity and specificity to investigate PE incidence. In conclusion, prevention and control of COVID-19 remains paramount in the current pandemic, but repeated assessment and optimal management of PE complications may significantly modify the prognosis and reduce mortality in patients with COVID-19 [24].

## Data Availability

Not applicable.

## Abbreviations

PE: Pulmonary embolism
CI: Confidence interval
COVID-19: Coronavirus disease 2019

## References

[1] Susen S, Tacquard CA, Godon A, Mansour A, Garrigue D, Nguyen P (2020). Prevention of thrombotic risk in hospitalized patients with COVID-19 and hemostasis monitoring. Crit Care.

[2] Wang Y, Zhang D, Du G, Du R, Zhao J, Jin Y (2020). Remdesivir in adults with severe COVID-19: a randomised, double-blind, placebo-controlled, multicentre trial. Lancet.

[3] Riker RR, May TL, Fraser GL, Gagnon DJ, Bandara M, Zemrak WR (2020). Heparin-induced Thrombocytopenia with Thrombosis in COVID-19 Adult Respiratory Distress Syndrome. Res Pract Thromb Haemost..

[4] LeBrun DG, Konnaris MA, Ghahramani GC, Premkumar A, DeFrancesco CJ, Gruskay JA (2020). Hip Fracture Outcomes During the COVID-19 Pandemic: Early Results From New York. J Orthop Trauma.

[5] Wichmann D, Sperhake JP, Lütgehetmann M, Steurer S, Edler C, Heinemann A, et al. Autopsy Findings and Venous Thromboembolism in Patients With COVID-19. Ann Intern Med. 2020;M20-2003. 10.7326/M20-2003 PMC7240772 32374815

[6] Klok FA, Kruip MJHA, van der Meer NJM, Arbous MS, Gommers D, Kant KM (2020). Confirmation of the high cumulative incidence of thrombotic complications in critically ill ICU patients with COVID-19: An updated analysis. Thromb Res..

[7] Llitjos JF, Leclerc M, Chochois C, Monsallier JM, Ramakers M, Auvray M (2020). High incidence of venous thromboembolic events in anticoagulated severe COVID-19 patients. J Thromb Haemost.

[8] Helms J, Tacquard C, Severac F, Leonard-Lorant I, Ohana M, Delabranche X (2020). High risk of thrombosis in patients with severe SARS-CoV-2 infection: a multicenter prospective cohort study. Intensive Care Med.

[9] Menter T, Haslbauer JD, Nienhold R, Savic S, Deigendesch H, Frank S (2020). Postmortem examination of COVID-19 patients reveals diffuse alveolar damage with severe capillary congestion and variegated findings in lungs and other organs suggesting vascular dysfunction. Histopathology.

[10] Bompard F, Monnier H, Saab I, Tordjman M, Abdoul H, Fournier L, et al. Pulmonary embolism in patients with Covid-19 pneumonia. Eur Respir J. 2020;2001365. 10.1183/13993003.01365-2020 PMC7236820 32398297

[11] Hékimian G, Lebreton G, Bréchot N, Luyt CE, Schmidt M, Combes A (2020). Severe pulmonary embolism in COVID-19 patients: a call for increased awareness. Crit Care.

[12] Artifoni M, Danic G, Gautier G, Gicquel P, Boutoille D, Raffi F (2020). Systematic assessment of venous thromboembolism in COVID 19 patients receiving thromboprophylaxis: incidence and role of D dimer as predictive factors. Journal of Thrombosis and Thrombolysis.

[13] Fraissé M, Logre E, Pajot O, Mentec H, Plantefève G, Contou D (2020). Thrombotic and hemorrhagic events in critically ill COVID-19 patients: a French monocenter retrospective study. Crit Care.

[14] Thomas W, Varley J, Johnston A, Symington E, Robinson M, Sheares K (2020). Thrombotic complications of patients admitted to intensive care with COVID-19 at a teaching hospital in the United Kingdom. Thromb Res..

[15] Lodigiani C, Iapichino G, Carenzo L, Cecconi M, Ferrazzi P, Sebastian T (2020). Venous and arterial thromboembolic complications in COVID-19 patients admitted to an academic hospital in Milan. Italy. Thromb Res..

[16] Poissy J, Goutay J, Caplan M, Parmentier E, Duburcq T, Lassalle F (2020). Pulmonary Embolism in Patients With COVID-19: Awareness of an increased prevalence. Circulation.

[17] Gervaise A, Bouzad C, Peroux E, Helissey C. Acute pulmonary embolism in non-hospitalized COVID-19 patients referred to CTPA by emergency department. Eur Radiol. 2020;1-8. 10.1007/s00330-020-06977-5 PMC7280685 32518989

[18] Longchamp A, Longchamp J, Manzocchi‐Besson S, Whiting L, Haller C, Jeanneret S (2020). Venous thromboembolism in critically Ill patients with COVID‐19: Results of a screening study for deep vein thrombosis. Res Pract Thromb Haemost.

[19] Leonard-Lorant I, Delabranche X, Severac F, Helms J, Pauzet C, Collange O, et al. Acute pulmonary embolism in COVID-19 patients on CT angiography and relationship to D-dimer levels. Radiology. 2020;201561. 10.1148/radiol.2020201561 PMC7233397 32324102

[20] Grillet F, Behr J, Calame P, Aubry S, Delabrousse E. Acute pulmonary embolism associated with COVID-19 pneumonia detected by pulmonary CT angiography. Radiology. 2020;201544. 10.1148/radiol.2020201544 PMC7233384 32324103

[21] Rothberg MB, Haessler SD (2010). Complications of seasonal and pandemic influenza. Crit Care Med.

[22] Bikdeli B, Wang Y, Jimenez D, Parikh SA, Monreal M, Goldhaber SZ (2019). Pulmonary embolism hospitalization, readmission, and mortality rates in US older adults, 1999-2015. JAMA.

[23] Mojoli F, Mongodi S, Orlando A, Arisi E, Pozzi M, Civardi L (2020). Our recommendations for acute management of COVID-19. Crit Care.

[24] Zhai Z, Li C, Chen Y, Gerotziafas G, Zhang Z, Wan J (2020). Prevention and treatment of venous thromboembolism associated with coronavirus disease 2019 infection: a consensus statement before guidelines. Thromb Haemost.

